# Re-examining tau-immunoreactive pathology in the population: granulovacuolar degeneration and neurofibrillary tangles

**DOI:** 10.1186/s13195-015-0141-2

**Published:** 2015-08-28

**Authors:** Sally Hunter, Thais Minett, Tuomo Polvikoski, Elizabeta Mukaetova-Ladinska, Carol Brayne, the Cambridge City over-75s Cohort Collaboration

**Affiliations:** Department of Public Health and Primary Care, Institute of Public Health, Forvie Site, University of Cambridge School of Clinical Medicine, Box 113 Cambridge Biomedical Campus, Cambridge, CB2 0SP UK; Department of Radiology, University of Cambridge School of Clinical Medicine, Box 218, Cambridge Biomedical Campus, Cambridge, CB2 0QQ UK; Institute of Neuroscience, Henry Wellcome Building for Neuroecology, Newcastle University, Framlington Place, Newcastle upon Tyne, NE2 4HH UK

## Abstract

**Introduction:**

Alzheimer’s disease (AD) is associated with neurofibrillary pathology, including neurofibrillary tangles (NFT), neuritic plaques (NP) and neuropil threads containing aggregated microtubule associated protein tau. Aggregated tau is also associated with granulovacuolar degeneration (GVD). The relationships between tau, GVD, NFT and dementia are unclear.

**Methods:**

We assessed hippocampal (CA1) tau-immunoreactive GVD and NFT pathology in brain donations from the population-representative Cambridge City over 75s Cohort (CC75C) using the CERAD protocol and a modified protocol that included a morphological characterisation of tau-immunoreactive deposits within neurons as NFTs or as GVD. Associations between GVD, NFT and dementia were investigated.

**Results:**

Hippocampal pyramidal neurons affected with either NFT or GVD are common in the older population. Some tau-immunoreactive deposits resemble ghost GVD neurons. Tau immunoreactivity identified GVD in 95 % cases rated as none with haematoxylin and eosin staining. Both severe NFT (odds ratio (OR) 7.33, 95 % confidence interval (CI) 2.01; 26.80, *p* = 0.003) and severe GVD (OR 7.48, 95 %(CI) 1.54; 36.24, *p* = 0.012) were associated with dementia status. Increasing NFT (OR 2.47 95 %(CI) 1.45; 4.22, *p* = 0.001) and GVD (OR 2.12 95 %(CI) 1.23; 3.64, *p* = 0.007) severities are associated with increasing dementia severity. However, when the analyses were controlled for other neuropathologies (NFT, NP, Tar-DNA binding Protein-43 and amyloid deposits), the associations between GVD and dementia lost significance.

**Conclusions:**

Current neuropathological assessments do not adequately evaluate the presence and severity of the GVD pathology and its contribution to dementia remains unclear. We recommend that protocols to assess GVD should be developed for routine use and that tau, in a non-PHF associated conformation, is reliably associated with GVD.

**Electronic supplementary material:**

The online version of this article (doi:10.1186/s13195-015-0141-2) contains supplementary material, which is available to authorized users.

## Introduction

Alzheimer’s disease (AD) is characterised clinically by memory loss, cognitive impairment and behavioural problems [1], and neuropathologically by neuronal and synaptic loss and by various deposits containing the amyloid-beta protein (Aβ) and aggregated microtubule-associated protein tau in the form of neurofibrillary pathologies including neurofibrillary tangles (NFT), dystrophic neurites in neuritic plaques (NP) and neuropil threads (NT) [2]. Assessments of these lesions form the basis for the neuropathological confirmation of a clinical diagnosis of probable AD according to the Consortium to Establish a Registry for Alzheimer’s Disease (CERAD) protocol [2, 3] and the assessment of tau-immunoreactive (IR) changes according to Braak stage [4, 5]. The assessments for neuropathologically diagnosing AD have been updated recently to include assessments of Aβ deposits [6, 7].

The tau-associated pathologies, visualised by various methods and assessed using both the CERAD protocol [2, 3] and Braak staging [5, 8], include NFT, NP and NT. NFT are intracellular fibrous inclusions composed of paired helical filaments (PHFs) of aggregated and often hyperphosphorylated tau [9]. The CERAD protocol [2, 3] counts only those neurons with mature, robustly staining NFT; those neurons with diffuse or granular staining are classified as pre-tangles and are not counted. The morphology of tau-IR cytoplasmic inclusions is not considered. The presence of extracellular ghost NFT, the remains of NFT after the neuron has died, are noted in both CERAD and Braak staging. These are assessed on haematoxylin and eosin (H & E) or silver stained slides because many tau-IR epitopes associated with NFT are lost [10].

In addition to the well-recognised AD-associated pathologies and argyrophilic grains in argyrophilic grain disease (AGD) [11], various studies have demonstrated that the dense granules of granulovacuolar degeneration (GVD) also react with tau antibodies directed against various epitopes [10, 12–15] but not those directed against some conformational epitopes specific for PHFs [16, 17]. The possibility of potential confusion relating to the interpretation of tau-IR pathology has been noted previously [15]. GVD, first described by Teofil Simchowicz [18], is characterised by neurons with two or more double membrane-bound cytoplasmic vacuoles containing an electron-dense granule [19] and are usually assessed on H & E slides. GVD is noted in the CERAD protocol as present or absent in the hippocampus [2, 3]. GVD is distinct from other tau-IR granules described previously that lack membrane enclosed vacuoles and have been interpreted as PHF core bodies [20].

GVD within hippocampal pyramidal cells has previously been associated with increasing age and AD [21–23] and is associated with intraneuronal accumulation of tau protein [21]. GVD is also found in other neurodegenerative disorders, including Pick’s disease, multiple system atrophy with Parkinsonism, amyotrophic lateral sclerosis with dementia, Down syndrome and progressive supranuclear palsy [15, 24, 25]. Previous work has suggested a possible progression of development for GVD [23, 26], ranging from a few cytoplasmic granulovacuolar inclusions to neurons completely filled with granulovacuolar inclusions, which may follow topographical stages [15, 27]. While the association of GVD with AD has long been noted [12, 21], a recent study estimating the contributions of rare or disregarded pathologies [28] found no significant association between GVD and dementia when plaques and tangles were controlled for—raising questions relating to the significance of GVD to dementia.

The literature reviewed suggests that the relationship between GVD and dementia is unclear and that there is potential for confusion in interpretations of tau-IR pathologies. We examined the pathology of tau-IR GVD and NFT in detail in the cornus ammonis (CA) fields of the hippocampus in the population-based Cambridge City over-75s Cohort (CC75C) with an antibody against the C-terminal portion of tau that is not dependent on phosphorylation or PHF-specific epitopes. We categorised neuronal tau pathology as NFT, GVD or mixed (NFT+GVD), and investigated how this detailed morphology relates to current assessments of GVD and tau pathology according to well-accepted protocols and relates to the associations between NFT, GVD and dementia.

## Materials and methods

### The study sample

CC75C is a population-based, longitudinal study of aging and dementia with a brain donation programme [29]. Each study participant gave consent to participate and for brain donation. Consent for brain donation was also given by next of kin. Each phase of the CC75C study has been approved by the Cambridge Research Ethics Committee.

At the time of this study a total of 237 brain donations had CERAD measures, of which 211 cases were included for all analyses. Twenty five slides were not available or the area of interest was not present on the slide to score tau-IR GVD, NFT and GVD+NFT. For one slide, CA1 was not evaluable owing to complete neuronal loss.

A consensus diagnosis for dementia status at death consistent with *Diagnostic and Statistical Manual of Mental Disorders* 4th edition (DSM-IV) criteria [30] was agreed by clinicians, blinded to neuropathology reports, using post-mortem review of all clinical information including proxy informant data, death certificates and retrospective informant data after death. Dementia severity was rated as none, minimal, mild, moderate and severe [31]. Where dementia severity could not be rated it was scored as unknown.

### Neuropathological protocols

Brain collection for this study occurred between 1989 and 2009. After death, the brains were removed as soon as possible in the local mortuary. The brains were bisected in the sagittal plane. One cerebral hemisphere was dissected coronally into approximately 1 cm slices, macroscopically examined and then snap-frozen to –80 °C. The other half of the brain was fixed in formalin for at least 6 weeks and dissected coronally into approximately 1 cm slices. For diagnostic purposes, tissue blocks for paraffin embedding were taken from the hippocampus (at the level of the lateral geniculate body), entorhinal cortex (at the level of the mammillary body), frontal, temporal, parietal and occipital lobes, basal ganglia, thalamus, pons, medulla, cerebellum and two levels of the midbrain. Serial sections from the paraffin-embedded brain tissue samples were assessed for neuropathology blind to clinical status according to the CERAD protocol [2, 3] and Braak stage [4, 5]. All slides were produced by the Cambridge Brain Bank, Cambridge, UK and assessments were performed blind to clinical status by neuropathologists at Addenbrooke’s Hospital, Cambridge, UK. All immunostained sections for diagnosis were counterstained with Ehrlich’s haematoxylin with diaminobenzidine as the chromagen.

Lewy bodies were assessed as present or absent using 10 μm sections stained with H & E in combination with slides stained with anti-ubiquitin antibody (pAb BR 251, Z0458; DAKO, Glostrup, Denmark) (first 174 donations) or with anti-α-synuclein (SA3400; Biomol International, Enzo Life Sciences, Farmingdale, New York, USA) (last 63 cases). Aβ deposits as senile plaques and cerebral amyloid angiopathy (CAA) were visualised with Congo red and/or anti-Aβ antibody (M872, Clone 6 F/3D; DAKO) on 10 μm sections and assessed as none, mild, moderate or severe according to the CERAD protocol [2].

Inclusions reactive with tar-DNA binding protein 43 (TDP-43) were assessed using 9 μm sections from the hippocampus and entorhinal cortex stained with anti-TDP-43 antibody (pS409/410-2; Cosmo Bio Co. Ltd, Tokyo, Japan). Slides were counterstained with Harris’ haematoxylin with diaminobenzidine as the chromagen. Solid neuronal inclusions were assessed, based on the protocol in Neumann et al. [32], as none (no inclusions), minimal (one inclusion per slide), mild (one or more inclusions in up to half the fields of view per slide), moderate (a few inclusions in over half the fields of view per slide) or severe (a few inclusions in most fields of view per slide).

Sections 10 μm thick from the hippocampus and entorhinal cortex were immunostained with anti-tau monoclonal antibody (mAb) 11.57 (supplied by The Cambridge Brain Bank, dilution 1:5; gift donated by Professor Claude Wischik, University of Aberdeen, UK) to visualise NFT, NP and dystrophic neurites. mAb 11.57 was raised against a pronase-treated PHF core sub-fraction and recognises a phosphorylation and conformation-independent epitope in the C-terminal region of tau [12]. mAb 11.57 recognises NFT, NP, NT and GVD bodies and the grains of AGD but not ghost NFT [12, 33]. During the diagnostic process, an experienced neuropathologist scored the severity of NFT according to the CERAD protocol [2, 3] as either none, mild, moderate or severe. GVD, defined as neurons with two or more membrane-bound cytoplasmic vacuoles containing a basophilic granule, was rated according to the CERAD protocol [2, 3] as present or absent on hippocampal sections 10 μm thick stained with H & E.

A second protocol was designed to assess the separate tau-IR pathologies based on morphology. Tau-IR NFT (defined as neurons with fibrous deposits of tau and no vacuoles containing an electron dense granule), tau-IR GVD (defined as neurons with two or more membrane-bound cytoplasmic vacuoles containing a tau-IR dense granule and no fibres) and neurons containing mixed pathology with both fibrous and granulovacuolar pathology (NFT+GVD) were scored at 100× magnification as none, an isolated example per area, and as mild, moderate or severe by comparison with the references images for NFT from the CERAD protocol [3]. The same slides were assessed with both protocols. Higher magnifications (200× and 400×) were used to assess the detailed morphology of any tau-IR structures that could not be classified at lower magnification (100×). Separate scores for the hippocampal regions CA1 and CA2–3, the regions most associated with tau immunoreactivity, were generated by SH and inter-rated by TP. Regions CA2 and CA3 were combined for scoring owing to difficulties in reliably defining the CA2–CA3 boundary. Photomicrographs were taken with a Leica DM LB microscope with a DC500 digital camera Wetzlar, Germany.

### Inter-rater analysis

The extent of agreement between two raters (SH and TP) in relation to severity of NFT, GVD and NFT+GVD in CA1 and CA2–3 was assessed by calculating Gwet’s AC2 coefficients (Table 1). This is a paradox-resistant alternative to Kappa’s coefficient when the overall percentage agreement is high [34]. The coefficient calculations were performed using Agreestat 2011.2 (Advanced Analytics, Gaithersburg, MD, USA). The extent of agreement was assessed using the benchmark proposed by Landis and Koch [35]; a coefficient >0.6 indicates substantial agreement and a value >0.8 near-perfect agreement. In general, NFT and GVD were seen to have AC2 coefficients >0.6, implying substantial agreement, which was more marked in CA1 (Table 1). The only exception was for NFT+GVD in CA2–3 (AC2 = 0.13), where agreement was poor. This was most probably due to a combination of differences in assigning the CA1/CA2 boundary and the rarity of neurons with both tau-IR NFT and GVD pathologies. Data for NFT+GVD were not included in further analyses.

**Table 1:** Inter-rater reliability on tau-IR GVD, NFT and GVD+NFT by hippocampal area

	AC2	SE	95 % CI (AC2)	*p* value
CA2–3				
NFT	0.73	0.11	0.51–0.95	<0.001
NFT+GVD	0.13	0.22	−0.33 to 0.58	0.568
GVD	0.67	0.09	0.49–0.85	<0.001
CA1				
NFT	0.89	0.06	0.77–1.00	<0.001
NFT+GVD	0.76	0.09	0.57–0.96	<0.001
GVD	0.76	0.08	0.60–0.92	<0.001

*AC2* Gwet’s coefficient, *CA* cornus ammonis, *CI* confidence interval, *GVD* granulovacuolar degeneration, *NFT* neurofibrillary tangles, *SE* standard error

### Statistical analysis

Inter-rater reliability was not substantial for NFT+GVD in CA2–3, so further analyses were restricted to NFT and GVD in the CA1 area only. The mean (standard deviation) is presented when applicable.

The relationships between tau-IR GVD and NFT with dementia status were verified using logistic regression. Dementia status was defined as the dependent variable and tau-IR scores in CA1 as independent variables. Three hierarchical models were created: model 1 controlled for sex and age at death; model 2 was additionally controlled for NFT and NP; and model 3 additionally controlled for TDP-43 and amyloid deposits. The same approach was used to verify the relationships between tau-IR GVD and NFT with dementia severity, using ordinal logistic regression.

The Spearman coefficient (*r*) was calculated to verify the relationships between different pathologies and different protocols. All tests were two-tailed. Statistical analyses were performed using statistical package STATA, version 12 College Station, Texas, USA.

## Results

In total, 211 brains were included in the sample. Among them, 147 (70 %) were women. Mean age of death was 90.97 (4.51) years. Dementia status at death could not be determined in eight (4 %) participants, 71 (34 %) did not have dementia and 132 (63 %) had dementia at death.

Both NFT and GVD were common; using the new protocol, 91 % of the cases surveyed showed both GVD and NFT in the hippocampal CA1 fields, 1 % had NFT with no GVD, 4 % had GVD with no NFT and 4 % had neither NFT nor GVD pathology.

### Relationship between CERAD assessments and the new protocol

Table 2 presents a comparison between CERAD protocol GVD scores (columns) and the morphologically separated tau-IR GVD scores from the modified protocol (rows). Assessments using H & E slides underestimated GVD pathology: 95 % (54/57) of cases scored as having no GVD according to the CERAD protocol were scored as having at least mild GVD in CA1 according to the tau-IR stain.

**Table 2:** Comparison of GVD scores from the two protocols

	CERAD protocol GVD score	
Tau-IR GVD score^a^	No	Yes
No	3 (5)	1 (1)
Mild	21 (37)	18 (26)
Moderate	21 (37)	31 (45)
Severe	12 (21)	19 (28)

Data presented as *n* (%). Some GVD scores were marked as missing from the CERAD assessment, and therefore data are for cases which did have CERAD GVD scores (*n* = 126)

^a^From the modified protocol

*CERAD* Consortium to Establish a Registry for Alzheimer disease, *GVD* granulovacuolar degeneration, *IR* immunoreactive

Table 3 compares CERAD scores for NFT with those from the new protocol. Even though both the CERAD protocol and our modified tau-IR protocol scores represent the CA1 area, the correlation between those NFT scores was moderate (*n* = 126, *r* = 0.67, *p* <0.001).

**Table 3:** Comparison of NFT scores from the two protocols

	CERAD protocol NFT score			
Tau-IR NFT score^a^	No	Mild	Moderate	Severe
No	2 (67)	8 (17)	4 (5)	2 (3)
Mild	1 (33)	31 (67)	26 (32)	3 (4)
Moderate	0 (0)	7 (15)	40 (49)	27 (34)
Severe	0 (0)	0 (0)	11 (14)	48 (60)

Data presented as *n* (%). One score for NFT was marked as missing, and therefore data are for cases which did have CERAD NFT scores (*n* = 210)

^a^From the modified protocol

*CERAD* Consortium to Establish a Registry for Alzheimer disease, *IR* immunoreactive, *NFT* neurofibrillary tangles

### Relationship between NFT and GVD

Table 4 compares the scores for NFT and GVD according to the CERAD protocol assessments.

**Table 4:** Relationship between CERAD scores for NFT and GVD

	CERAD protocol NFT score			
CERAD protocol GVD score	No	Mild	Moderate	Severe
No	1 (100)	15 (43)	18 (43)	23 (48)
Yes	0 (0)	20 (57)	24 (57)	25 (52)

Data presented as *n* (%)

*CERAD* Consortium to Establish a Registry for Alzheimer disease, *GVD* granulovacuolar degeneration, *NFT* neurofibrillary tangles

There was no significant correlation between NFT and GVD assessed with the CERAD protocol (*n* = 126, *r* = −0.03, *p* = 0.760).

Table 5 compares the scores for NFT and GVD according to the new tau-IR protocol.

**Table 5:** Relationship between new protocol scores for NFT and GVD

	NFT score from tau-IR protocol			
GVD score from tau-IR protocol	No	Mild	Moderate	Severe
No	8 (50)	1 (2)	0 (0)	2 (3)
Mild	7 (44)	38 (62)	9 (12)	3 (5)
Moderate	1 (6)	22 (36)	45 (60)	21 (36)
Severe	0 (0)	0 (0)	21 (28)	33 (56)

Data presented as *n* (%)

*GVD* granulovacuolar degeneration, *IR* immunoreactive, *NFT* neurofibrillary tangles

There was moderate correlation between NFT and GVD using the new tau-IR protocol (*n* = 211, *r* = 0.65, *p* <0.001).

### Relationships between dementia and tau-IR pathologies

Analyses for tau-IR were conducted on 211 brain donations. Only two cases with hippocampal Lewy body pathology and only a few cases with moderate/severe CAA were identified; these neuropathologies were therefore not included in further analyses. The distributions between dementia severity and various hippocampal neuropathologies are shown in Additional file 1.

The relationship between dementia and tau-IR GVD was verified by logistic regression with dementia status as the dependent variable and tau-IR GVD in CA1 status as the independent variable. The analysis was controlled by sex and age at death in model 1 (Table 6). Severe tau-IR GVD was significantly related to dementia regardless of sex and age of death; however, when the analyses were also controlled by NFT and NP (Table 6, model 2), the relationship between dementia and severe tau-IR GVD lost significance. The loss of significance remained when hippocampal TDP-43 and amyloid deposits (see Additional file 1) were controlled for (Table 6, model 3).

**Table 6:** Logistic regression models showing the relationships between hippocampal pathologies and presence of clinical dementia

	Model 1			Model 2			Model 3		
	OR	CI	*p* value	OR	CI	*p* value	OR	CI	*p* value
GVD-IR									
Mild	1.04	(0.25–4.38)	0.956	1.00	(0.23–4.35)	1.000	1.00	(0.22–4.52)	0.998
Moderate	1.55	(0.38–6.31)	0.542	1.07	(0.24–4.76)	0.931	1.03	(0.22–4.80)	0.972
Severe	7.48	(1.54–36.24)	0.012	3.90	(0.71–21.45)	0.118	3.81	(0.65–22.41)	0.139
NFT-IR									
Mild	1.21	(0.37–3.93)	0.753	1.26	(0.38–4.25)	0.705	1.39	(0.36–5.45)	0.635
Moderate	2.27	(0.70–7.31)	0.170	1.66	(0.46–5.98)	0.435	2.61	(0.61–11.19)	0.198
Severe	7.33	(2.01–26.80)	0.003	4.86	(1.17–20.28)	0.030	9.99	(1.97–50.54)	0.005

Model 1 controlling for age and sex; model 2 additionally controlling for NFT and NP; model 3 additionally controlling for TDP-43 and amyloid deposits

*CI* confidence interval, *GVD* granulovacuolar degeneration, *IR* immunoreactive, *NFT* neurofibrillary tangles, *NP* neuritic plaques, *OR* odds ratio, *TDP-43* Tar-DNA binding protein 43

The relationships between dementia severity and severity of tau-IR GVD and NFT were tested using ordinal logistic regressions controlling for sex and age at death in model 1 (Table 7). For these analyses, tau-IR scores were dichotomised into none/isolated/mild vs. moderate/severe. Increasing tau-IR GVD and tau-IR NFT severities were significantly associated with increasing dementia severity (Table 7, model 1). However, when the analysis was also controlled by NFT and NP (see Additional file 1), the relationship between dementia severity and tau-IR GVD was weaker and lost significance (Table 6, model 2). The loss of significance remained when hippocampal TDP-43 and amyloid deposits (see Additional file 1) were controlled for (Table 6, model 3).

**Table 7:** Ordinal logistic regression models showing the relationships between hippocampal pathologies and severity of clinical dementia

	Model 1			Model 2			Model 3		
	OR	CI	*p* value	OR	CI	*p* value	OR	CI	*p* value
GVD-IR	2.12	(1.23–3.64)	0.007	1.30	(0.69–2.44)	0.422	1.31	(0.68–2.54)	0.417
NFT-IR	2.47	(1.45–4.22)	0.001	1.61	(0.84–3.10)	0.152	3.16	(1.48–6.71)	0.003

Model 1 controlling for age and sex; model 2 additionally controlling for NFT and NP; model 3 additionally controlling for TDP-43 and amyloid deposits

*CI* confidence interval, *GVD* granulovacuolar degeneration, *IR* immunoreactive, *NFT* neurofibrillary tangles, *NP* neuritic plaques, *OR* odds ratio, *TDP-43* Tar-DNA binding protein 43

### Descriptions of tau-IR pathology

**Fig. 1:**
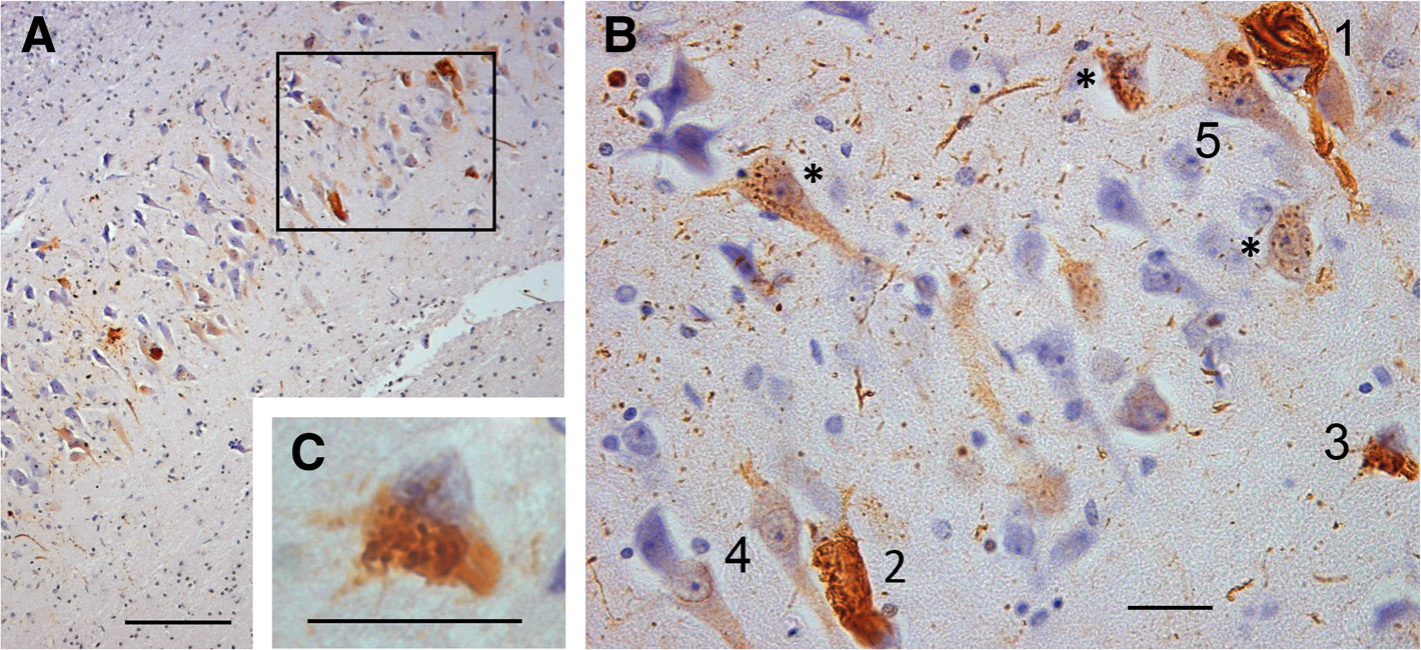
GVD and NFT at the CA2–CA1 boundary. **a** CA2–CA1 boundary at 159× magnification; scale bar = 100 μm. **b** Marked area in A at 638× magnification; scale bar = 20 μm: 1 NFT bearing neuron, 2, 3 neurons with both GVD granules and fibrils that under low power in A could be mistaken for ‘pure’ NFT, 4 two neurons with diffuse tau staining lacking any clear morphology and showing a clear hard line around the nucleus, 5 a neuron with GVD also containing two larger discrete fibrous bundles, *examples of neurons with GVD. **c** The cell marked 3 at 1600× magnification showing GVD and fibrils; scale bar = 20 μm. *CA* cornus ammonis, *GVD* granulovacuolar degeneration, *NFT* neurofibrillary tangles

Hippocampal pyramidal neurons can develop NFT, GVD or, less commonly, co-existing GVD and NFT as illustrated in Fig. 1.

**Fig. 2:**
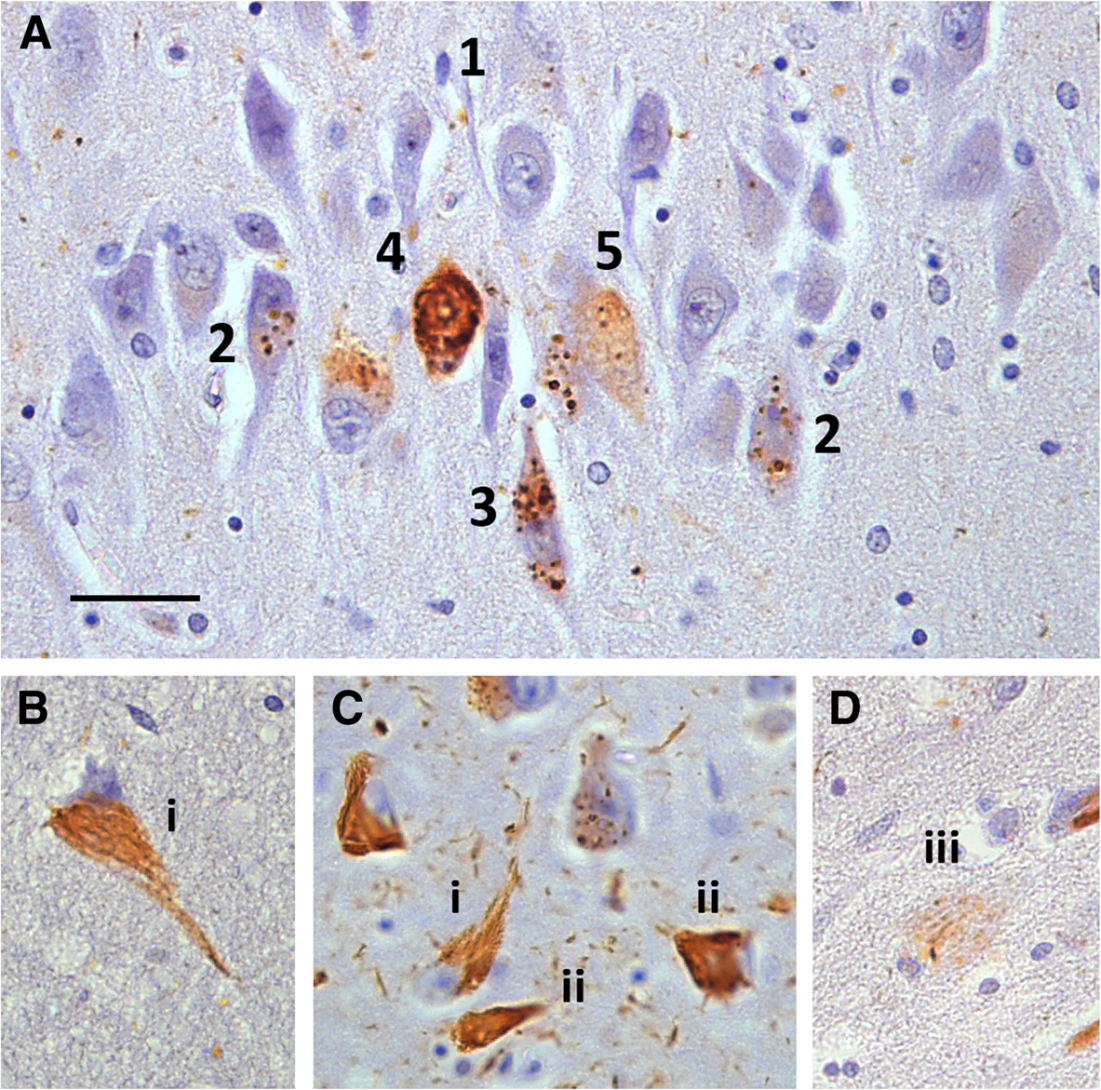
GVD and NFT progression in the hippocampal CA1 field. **a** Neurons in CA1 at 638× magnification showing various stages of GVD pathology: 1 a few granules in the neuronal cytoplasm staining weakly and one granule not at all for tau, 2, 3 more cytoplasmic granules develop and tau staining increases in intensity, 4 the cell body is completely filled with strongly staining small GVD and could be mistaken for NFT, 5 the neuron appears distended and the cell body is filled with large GVD with distended vacuoles. **b**, **c**, **d** CA1 neurons showing NFT progression at 638× magnification: i neurons showing a possible early stage of NFT formation with lightly staining PHF fibres, ii neurons showing robust tau staining of fibrous PHFs, iii ghost tangle. Scale bar = 20 μm. *CA* cornus ammonis, *GVD* granulovacuolar degeneration, *NFT* neurofibrillary tangles, *PHF* paired helical filament

In Fig. 2a, GVD granules in neurons can be seen to range from a few weakly staining, and very rarely, non-staining granules within a neuron to an increasing number of membrane-enclosed granules until the whole cell body is filled with intensely staining GVD. Distended neuronal bodies with large swollen GVD bodies can also be seen (Fig. 2a, neuron 5). This can be compared with the well-accepted progression in NFT ranging from a few lightly stained cytoplasmic filaments (Fig. 2b, c, neurons i) to robustly staining mature fibrous NFT (Fig. 2b, c, neurons ii), with ghost NFT as an end stage (Fig. 2d, area iii). There is a possibility that advanced stages of GVD could be mistaken for NFT, as seen in Figs. 1 and 2. The CA2 area at low power in Fig. 1 appears to contain at least three neurons with robustly staining cytoplasmic inclusions, but at higher magnifications only one neuron from the marked area contains NFT, with others showing advanced GVD or a combination of GVD-like and fibrous inclusions.

Very rarely, neurons with GVD also contained one or more discrete fibrous bundles (Fig. 1b, neuron 5) that could not be classified as NFT or GVD/NFT mixed and so were not included in the assessments. Pyramidal neurons with diffuse cytoplasmic tau reactivity, often with a defined line around the nucleus (Fig. 1b, neurons 4) and previously described in association with AGD [36], seen in the hippocampal areas CA1, CA2 and CA3 were also not included in assessments for this study.

**Fig. 3:**
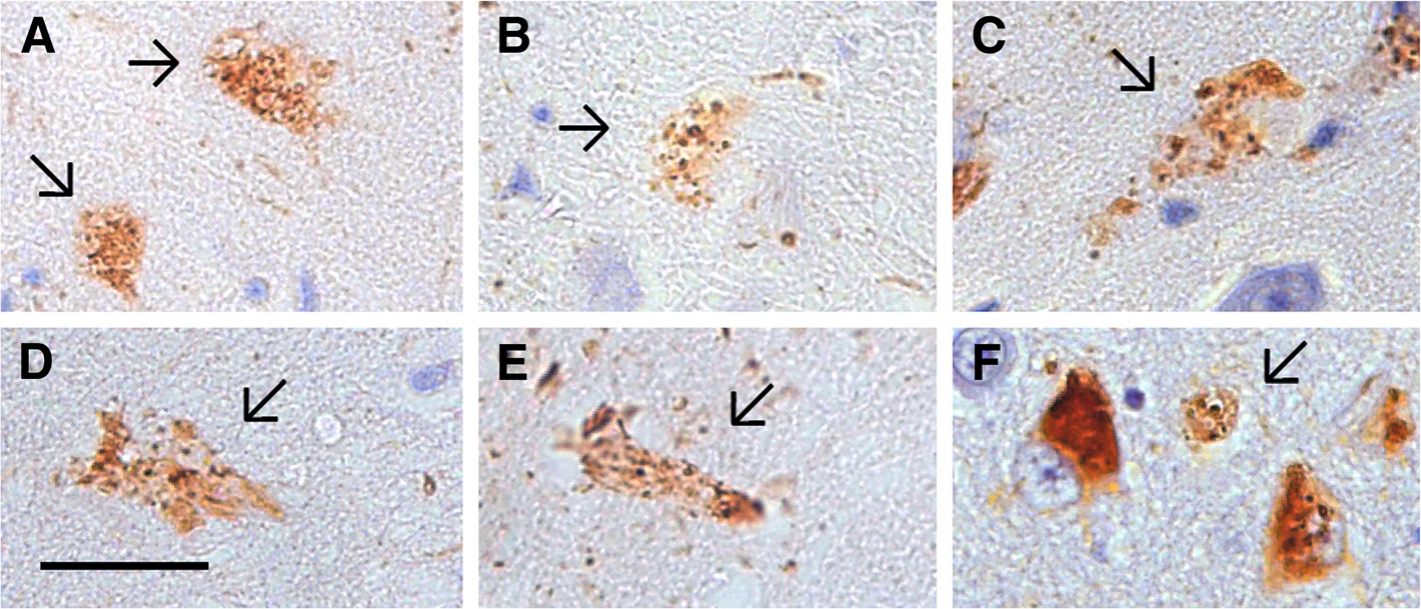
End stage ‘ghost’ GVD. **a**–**f** Examples of possible ‘ghost’ GVD. All panels at 638× magnification; scale bar = 20 μm. *Arrows*, deposits resembling neurons with GVD but with no discernible living cell body or nucleus. *GVD* granulovacuolar degeneration

In addition to the well-recognised ghost tangles associated with NFT, we also found what appeared to be extracellular ghost GVD, showing clear granulovacuolar inclusions but no evidence of a living neuron, examples of which are shown in Fig. 3. Unlike ghost NFT, these retained reactivity with mAb 11.57.

## Discussion

We found more GVD pathology using tau-IR than was detected by H & E, suggesting that GVD is missed with current protocols. The associations between dementia and both hippocampal (CA1) NFT and GVD are significant. Additionally, the severity of both NFT and GVD were significantly associated with dementia severity. However, the association between GVD and dementia lost significance when NFT, NP and other pathologies were controlled for.

We find that there is a potential for misinterpretation of tau pathology. Since many anti-tau antibodies react with both NFT and GVD, there is potential for neurons with GVD to be mistaken for pre-tangles and neurons with severe GVD to be mistaken for NFT at low magnifications. We also found tau-IR deposits resembling ghost GVD neurons that appeared morphologically different from ghost NFT.

This study has limitations. The selection of anti-tau immunostained slides used in this study was from the historical diagnostic collection and assessments were semi-quantitative and not stereological [37]; this may introduce bias because we assume a single slide will represent a specific case adequately. Since comparison of the scoring protocols required that we use the same diagnostic slides, missing slides—perhaps due to breakage over the ~25 years of the study to date—may introduce bias.

Our assessments of Lewy bodies are based on older protocols for 174/241 cases and therefore scores for Lewy bodies might be underestimates. Both hippocampal Lewy bodies and CAA were rare and not included in analyses.

In any study of this type, brains donated at death represent a cross-section of the cohort at a specific terminal point. While the presentation of GVD morphology in Fig. 2 appears to represent a progression of increasing severity, this is not certain. Additionally, the morphological similarity of structures in Fig. 3 to ghost GVD neurons is circumstantial. These interpretations would need to be confirmed by longitudinal studies.

Of the 211 cases included in our study, only 126 had measures for GVD from the CERAD protocol. It is possible that GVD was not seen on H & E staining and therefore no measure was recorded. Alternatively, historically GVD has not been prioritised as a dementia-related pathology and therefore GVD assessments may have been neglected. We did not use a second marker for GVD; however, we were very careful to count only neurons containing two or more structures with a dense granule surrounded by the halo of a vacuole and it is unlikely that we have overestimated GVD. A marker for GVD has yet to be validated and, given our poor correlations between GVD and NFT as assessed using the CERAD protocol, a consensus regarding the reliability of GVD markers is required.

Tau-IR reliably marks nearly all dense granules associated with GVD and we identified more cases with tau-IR GVD than with the standard assessment protocol using H & E staining. Similarly, a study using antibodies to tubulin found four times more GVD than H & E alone [38], and the phosphorylated ribosomal protein S6 (pS6) revealed more neurons containing both NFT and GVD than in our study [39], suggesting that GVD can be missed in standard neuropathological protocols using H & E. Thal et al. [15] suggest that tau is not a good marker for GVD owing to its involvement with various pathologies, and another candidate marker for GVD (pS6) revealed more neurons containing both NFT and GVD than in our study [39]; therefore it is possible that GVD-IR is masked by immunoreactivity for NFT and that our measures underestimate GVD when present in NFT.

Our more detailed assessment of tau-IR NFT correlated with that of the CERAD protocol moderately. The CERAD score represents the highest score measured over the entire hippocampal structure and our detailed score is for CA1 only. However, since CA1 is often the most severely affected hippocampal structure [4, 5], we would expect good correlation. If the morphology of tau-IR is not considered, there is potential for occasional neurons severely affected by GVD and neurons with both GVD and NFT to be misclassified as pure NFT, as illustrated in Figs. 1 and 2. This may also contribute to the moderate correlation found.

We found a significant association between both NFT and GVD with dementia. However, when NFT and NP were controlled for, the association between GVD and dementia lost significance. This is in agreement with results from the study by Keage et al. [28]. In agreement with Ball [40], our results suggest that GVD severity increases as NFT severity increases. A loss of significance can mean that GVD is not an independent contributor to dementia and so is ‘unimportant’ or that there is some mechanism involving GVD, NFT and plaques that undermines the assumption of independence for this statistical test. With increasing evidence that NFT and GVD share some features, including tau, and not others (e.g., TDP-43 [41]), it is possible that loss of significance reflects some shared process(es) rather than GVD not being an important contributor to dementia. At a mechanistic level, various studies imply that GVD is a pre-tangle and/or contributes to early NFT formation [42, 43]. This conflicts with other studies [12], including ours, which present evidence that GVD can be interpreted as a separate process.

Our estimates of the associations between both severities of GVD and NFT and dementia status may be unreliable because only CA1 scores were included. For a future detailed investigation of the relationships between NFT, GVD and dementia, neocortical and subcortical areas should be included [15].

In agreement with Bondareff et al. [12], in each available case from the CC75C cohort affected by both NFT and GVD we have observed neurons with classic fibrillar NFT only, classic GVD only and, more rarely, neurons with both NFT and GVD (Figs. 1 and 2). While these pathologies can rarely occur together in the same neuron, we do not see neurons in which GVD-like deposits can be interpreted as developing into NFT following the generally accepted progression from pre-tangle to mature NFT to ghost tangle as the CERAD protocol and other studies imply [42, 43]. Rather, NFT and GVD appear to have their own progression in severity (Fig. 2) and their own ghost forms (Figs. 2 and 3). Given the associations between GVD and NFT and dementia presented, our evidence supports previous suggestions that the formation of NFT and GVD are separate processes that can co-occur in any individual [23].

In comparison, the accepted longitudinal progression for NFT from pre-tangles to mature tangles to extracellular ghosts is also based on similar cross-sectional studies. Our findings suggest that the widely accepted progression of NFT may not be adequate to explain the variations in tau-IR we present. We suggest that the pathways of both granulovacuolar and neurofibrillary tau-IR pathology require careful evaluation to separate and understand the relationships between these two processes and their involvement in dementia. Additional processes may relate to diffuse tau-IR seen in neurons marked in Fig. 2 and these may be associated with AGD [36]. Definitions of what a pre-tangle is need clarification.

Most GVD granules are labelled strongly with tau, suggesting that tau is involved in the development of GVD at an early stage. GVD shows an amorphous structure on electron micrographs [14, 19], shows no fluorescence with thioflavin S [13] and is not marked by mAbs such as 7.51 [12], Alz-50 [25, 44], Tau-1 [17] and PHF-1 [16, 45] recognising epitopes specific to PHFs. The above evidence suggests that PHFs, typical of NFT, are not contained in GVD. Tau aggregation in GVD has been interpreted as representing a second pathway of tau aggregation [12]. Tau as a component of GVD will be missed when using PHF reactive antibodies. Why some neurons develop NFT, GVD or occasionally both is not known but could arise owing to different stress responses in different neuronal subtypes, different neurodegenerative processes or different stress responses within the neurons. Investigations into the initiation and development of GVD may offer alternative therapeutic targets.

## Conclusions

Tau in a non-PHF-associated conformation is reliably associated with GVD. Current neuropathological protocols do not adequately capture the extent of tau-IR pathology and require re-evaluation, in particular, to better assess the contribution of GVD to dementia and differentiate it from pathology of NFT. The relationships between NFT, GVD and pre-tangles are unclear, which has consequences for investigations into tau aggregation, especially at early stages of disease. We recommend that protocols to assess GVD should be developed for routine use.

## Additional file

Additional file 1: Table S1. Presenting the severity of hippocampal pathologies with dementia severity. *n* (row%); NFT-IR data from the new protocol; NFT (CERAD) data from the CERAD protocol. (DOC 80 kb)

## Abbreviations

Aβ: Amyloid-beta protein
AD: Alzheimer’s disease
AGD: Argyrophilic grain disease
CA: Cornus ammonis
CAA: Cerebral amyloid angiopathy
CC75C: Cambridge City over 75s Cohort
CERAD: Consortium to Establish a Registry for Alzheimer’s Disease
DSM-IV: *Diagnostic and Statistical Manual of Mental Disorders* 4th edition
GVD: Granulovacuolar degeneration
H & E: Haematoxylin and eosin
IR: Immunoreactive
mAb: Monoclonal antibody
NFT: Neurofibrillary tangles
NP: Neuritic plaques
NT: Neuropil threads
PHF: Paired helical filament
pS6: Phosphorylated ribosomal protein S6
TDP-43: Tar-DNA binding protein 43
