# Supplementary material for: Re-examining tau-immunoreactive pathology in the population: granulovacuolar degeneration and neurofibrillary tangles
Source: Alzheimers Res Ther. 2015 Aug 28;7(1):57. doi: 10.1186/s13195-015-0141-2 (PMC4551529; doi:10.1186/s13195-015-0141-2)
Supplement: Additional file 1: Table S1. — Presenting the severity of hippocampal pathologies with dementia severity. n (row%); NFT-IR data from the new protocol; NFT (CERAD) data from the CERAD protocol. (DOC 80 kb) [file 13195_2015_141_MOESM1_ESM.doc]

Supplementary Table 1. Distributions of severity of hippocampal pathologies with dementia severity

| Pathology | Severity | Dementia Severity n(%) | | | | | |
| --- | --- | --- | --- | --- | --- | --- | --- |
|  |  | None | MCI | Min | Mild | Mod | Sev |
| Amyloid plaques | No | 36(37) | 10(10) | 13(13) | 9(9) | 13(13) | 17(17) |
|  | Mild | 15(24) | 2(3) | 8(13) | 6(10) | 10(16) | 21(34) |
|  | Mod | 3(12) | 1(4) | 1(4) | 4(15) | 9(35) | 8(31) |
|  | Sev | 3(25) | 1(8) | 0(0) | 3(25) | 3(25) | 2(17) |
| CAA (meninges) | No | 48(30) | 14(9) | 18(11) | 17(11) | 26(16) | 36(23) |
|  | Mild | 8(27) | 0(0) | 3(10) | 3(10) | 5(17) | 11(37) |
|  | Mod | 1(13) | 0(0) | 1(13) | 2(25) | 3(38) | 1(13) |
|  | Sev | 0(0) | 0(0) | 0(0) | 0(0) | 1(50) | 1(50) |
| CAA (parenchymal) | No | 50(29) | 14(8) | 20(12) | 17(10) | 30(18) | 39(23) |
|  | Mild | 7(30) | 0(0) | 2(9) | 3(13) | 2(9) | 9(39) |
|  | Mod | 0(0) | 0(0) | 0(0) | 1(25) | 3(75) | 0(0) |
|  | Sev | 0(0) | 0(0) | 0(0) | 1(50) | 0(0) | 1(50) |
| GVD-IR | No | 5(50) | 1(10) | 2(20) | 0(0) | 1(10) | 1(10) |
|  | Mild | 21(38) | 6(11) | 6(11) | 6(11) | 8(14) | 9(16) |
|  | Mod | 26(31) | 6(7) | 10(12) | 8(10) | 12(14) | 21(25) |
|  | Sev | 5(10) | 1(2) | 4(8) | 9(17) | 15(29) | 18(35) |
| Lewy Bodies | No | 54(29) | 13(7) | 20(11) | 23(12) | 33(17) | 46(24) |
|  | Mild | 0(0) | 1(100) | 0(0) | 0(0) | 0(0) | 0(0) |
|  | Mod | 0(0) | 0(0) | 0(0) | 0(0) | 1(100) | 0(0) |
| Neuritic plaques | No | 30(54) | 6(11) | 6(11) | 5(9) | 5(9) | 4(7) |
|  | Mild | 17(25) | 4(6) | 8(12) | 8(12) | 13(19) | 18(26) |
|  | Mod | 9(14) | 4(6) | 7(11) | 8(13) | 14(22) | 22(34) |
|  | Sev | 1(8) | 0(0) | 1(8) | 2(17) | 3(25) | 5(42) |
| NFT  (CERAD) | No | 3(100) | 0(0) | 0(0) | 0(0) | 0(0) | 0(0) |
|  | Mild | 20(45) | 4(9) | 6(14) | 1(2) | 8(18) | 5(11) |
|  | Mod | 22(29) | 6(8) | 8(11) | 12(16) | 12(16) | 16(21) |
|  | Sev | 12(16) | 4(5) | 8(10) | 10(13) | 15(19) | 28(36) |
| NFT-IR | No | 7(47) | 1(7) | 1(7) | 0(0) | 2(13) | 4(27) |
|  | Mild | 24(41) | 6(10) | 7(12) | 7(12) | 6(10) | 9(15) |
|  | Mod | 19(28) | 6(9) | 5(7) | 8(12) | 17(25) | 13(19) |
|  | Sev | 7(12) | 1(2) | 9(15) | 8(14) | 11(19) | 23(39) |
| TDP-43 | No | 44(33) | 11(8) | 16(12) | 16(12) | 21(16) | 27(20) |
|  | Mild | 10(26) | 2(5) | 6(16) | 3(8) | 9(24) | 8(21) |
|  | Mod | 0(0) | 0(0) | 0(0) | 2(25) | 1(13) | 5(63) |
|  | Sev | 1(8) | 1(8) | 0(0) | 2(17) | 1(8) | 7(58) |

n(row%); NFT-IR data from the new protocol; NFT(CERAD) data from the CERAD protocol
